# Translations

**Published:** 1882-02

**Authors:** Seth N. Jordan

**Affiliations:** Columbus, Ga.


### Translations.

---

BY SETH N. JORDAN, M.D., COLUMBUS, GA.

---

*From Foreign Journals for the Atlanta Medical Register.*

---

*Diphtheria without Angina—Epidemic of Diphtheritic Paralysis. Boissaric. Gazette Hebdomedaire, Nos.* 21 and 22, 1881.—In an epidemic of diphtheria occupying narrow bounds and of a short duration of time, the author proved the following to be true: (1). Sudden appearance of paralysis without angina preceding, without any false membrane whatever, and which often resulted in death in a few hours or days; (2) in other cases, although ushered in by paralysis, diphtheritic angina developed later; (3)

in the midst of these singular phenomona, some cases ran the usual course of diphtheria without symptoms of paralysis either preceding or following. The author himself was attacked with the first form. We give his observations in as short space as possible: On the 18th of October he was consulted by a laborer, who lived in close proximity to the hospital, and who was suffering from a paralysis of the soft palate, but was still attending his duties. On examination of the throat not the slightest inflammation was observable. In the hospital B. found two patients, a young man 20 years old, and the wife of the steward, who presented like symptoms—nasal speech, difficulty in swallowing, etc.—and these, too, were not suffering from the slighest angina, nor was there any diphtheritic deposit present. Further investigations brought to light the fact, that just opposite the hospital, 100 metres distant, there were ten cases of diphtheria in one house; and these all had been accompanied by very violent symptoms. A child in this house fell sick on October 15th, with difficulty of speech and swallowing, and died in four days under heavy dyspnœa. The mother of this child, who had passed through child-birth two months previously, became ill on the 17th, and died on the 18th, in twenty-four hours after the beginning of the illness. From the commencement she was unable to swallow, her eyes remained closed, the pulse grew suddenly weak, and the respiration slow; in spite of all exertion, she was unable to expand the thorax. The grandmother of this woman was attacked with similar symptoms, and died in four days; no fever, no elevation of the temperature of the skin. This family was visited by relatives from a distance. The brother of the young woman came on the 16th, returned to his home on the 21st. He became sick on the 22d, showing the same symptoms, according to the report received from the physician who treated him, and died on the 27th. Finally a sister came on the 25th, developed a day later the disease, and died on the 28th. The father alone was spared an attack. As at first intimated, the disease spread to the neighboring houses from the patients at and near the hospital. The first mentioned patient, the laborer, grew worse in regard to the paralysis, and as he could not swallow, was nourished with beef enemata, receiving in addition 1 grm. of quinine subcutaneously. Ten days subsequent to the first appearance of the paralysis an unmistakable membrane developed in mouth and pharynx. With the appearance of this the symptoms of paralysis diminished. Patient could swallow beef tea with difficulty, which had previously been impossible. The membrane was thrown off on the 14th day. The paralysis which had also befallen the eyes and extremities, did not disappear before the lapse of two months. The author reports other similar cases at length. Other physicians in the same quarter had also many such cases as have been described, many ending in death. The author considers the disease analogous to the so-called scarlatina sine eruptio. The essence of diphtheria consists, in his opinion, not in deposits on, or in, the mucous membrane of the pharynx, as is the current opinion, but the diphtheritic poison can attack primarily the nerves, and especially those of the bulbus.

The epidemic was popularly attributed to poisoning from sardines, which, according to Gravis, Trousseau and others, do produce similar symptoms. On close investigation, however, it was clearly shown that only a very limited number of those affected had eaten sardines at any recent date.

---

*Operations for Fistulæ and Laparotomy. Zweifel. Wochenschrift, Berlin. No.* 22, 1881.—In Erlangen, for the last two years, Zweifel has made thirty three laparotomies and fifteen operations for fistula. Of twenty-one ovariotomies only one patient died of septic peritonitis, following the rupture of a suture during vomiting. Two died of tetanus. Two were attacked with parotitis.

Castration was performed twice on account of fibroids, once each on account of constant pain, congestion, swelling and neuralgia of both ovaries. Of these, two died, one from sepsis, the other ceased to menstruate, but died from a malignant tumor. Four hysterectomies were performed with complete success, all ending in recovery. Two operations performed after Freund's method, and three Cæsarean sections, resulted unfavorably. Two operations were given up, an exploratory incision proving in the first case the presence of primary carcinoma of the stomach; in the other, a dermoid tumor with obstinate adhesions. An operation for the removal of an extra-uterine fœtus resulted very favorably; the amnion had degenerated; it was removed after laparotomy. Of the patients with fistulæ, only one could be cured by a transverse obliteration of the vagina; two cases were rectal fistulæ.

In the Maternity, 77.7 per cent. of the labor cases were absolutely free of fever; 0.79 per cent. died of puerperal fever. One of these cases of death was referred directly to infection produced by an examination made by an interne, who, fifty-four hours previously, had made an autopsy.

---

*Antiseptic Suture of the Patella. Arch. fur Klin. Chirurgie, XXVI., p.* 286. *Pfeil Schneider.*—The author details a case of a fractured patella in a man 35 years old, which he treated with antiseptic sutures twenty-four hours after the injury. The result was complete recovery in forty-four days after the accident, with entire use of the knee from that day on.

The author found in literature nine similar cases of subcutaneous fracture of the patella in which the antiseptic suture was applied. A more exact analysis showed that the operation had been performed five times in old cases, and the other cases were in recent fractures, (1 refracture). No patient died. Six cases recovered without any reaction locally or generally having been noted. In one case the temperature went up to 103°; twice suppuration of the joint occurred. Bony union of the fragments was observed certainly eight times, and once probably (case of Lister). In the tenth case union was established by means of a short ligament (Cameron). In eight cases the joint remained movable; in the other two anchylosis was present before the operation. After mentioning the very favorable results obtained in such fractures in the pre-antiseptic period, the author arrives at the following conclusions: Antiseptic sutures in subcutaneous fracture of the patella is admissible, and is really benign in comparision to many operations that are carried out now-a-days with antiseptic precautions. He insists that strong sutures be used, and be allowed to remain untouched. The after-treatment consists in restitution of the knee joint; on this account, in favorable cases, patients must not remain more than two or three weeks in bed.

---

*Orange Colored Pus. Verneuil. Gazette Hebdom.* 1881, *No.* 18.—Orange colored suppuration, to which Delore was the first to call attention in 1854, has been seen by Verneuil only once, and that during 1871, at the time of the Commune, and in hospitals occupied by them. Then V. saw so many cases that he speaks of them as an epidemic. Broca endeavored to produce the micrococci in culture-fluid, but in vain. Verneuil states that this singularly colored pus in no case observed by him appeared before the third or fourth day after the injury was received. Its appearance is dependent on the kind of wound and the constitution of the patient. Generally the wound is contused, the gangrenous bits being held together by the orange-colored pus. Perfectly clean wounds never exhibit this peculiar color in the pus. In most cases the color disappears in a short time. As constitutional causes, Verneuil mentions alcoholism, diabetes mellitus and phosphaturia. Among other cases detailed fully, is one of morphineism. Delore held that the appearance of orange-colored pus demanded an unconditionally lethal prognosis; Verneuil, saw, however, a few cases recover. Delore, also, considered pyemia invariably the result of this pus. Verneuil did not observe this in every case. He believes there can be no prophylactic measures adopted against it.

---

*The Intra-Uterine Treatment of Puerperal Fever. Breisky. Zeitschrift,*1881, *I., p.* 316.—Breisky has treated fifteen cases of fever occurring in child-bed from infection, with permanent irrigation, usually beginning with a lukewarm two or three per cent. solution of carbolic acid, and gradually going down to ice water. Only in a few cases was any internal medicament ordered. In those cases in which the fever had already advanced, permanent irrigation influenced the course only when the affection of the genital canal was slight, and only in so far as the affected parts came in contact with the stream. The diminution of the temperature is evident. In phlegmone, peritonitis, parenchymatous infiltration of the uterus and adnexa, and in deep seated ulcerated ruptures, the employment of irrigation is of little avail. Still, B. considers it as an important factor with antipyretic remedies internally.

Those cases last mentioned demand imperatively accurate examination per vaginam before any local measures can be introduced. Breisky does not advocate the active method of prophylactic disinfection of the genital canal as a general measure His hospital for lying-in patients shows the following mortality: Mortality from puerperal fever reached from 6.07 in 1879, to 0.02 now; total mortality, 0.83.

---

*Eclampsia. H. Lohlein. Wochenschrift, No.* 40.—After having studied the annals of the Berlin Maternity, Königsberg Lying-in, and the Dresden and Berlin Charity hospitals, Lohlein is convinced that the prognosis of eclampsia is quite unfavorable ($\frac{1}{3}$ per cent. deaths.) He then considers the question of eclampsia puerperalis sine albuminuria. He rejects the idea that this form of the disease is something special, as epilepsy, for instance. The grounds advanced by the other side—the rarity of the trouble and the benign course—he does not consider proof. He is of the opinion that some of these cases are the result solely of pressure on the ureters during birth, and closure of the same. As to the period when the attacks occur, according to him, the nearer the attack is to the end of the birth, the better the prognosis, because in such cases the intoxication, at all events, is much less. In his remarks on prophylaxis, he lays particular stress on a favorable position—recommending lying on the side inclined to the abdomen. For the relief of existing convulsions, when albumen is present, he holds abortion to be indicated: (1) when cerebral symptoms of uremic intoxication are manifest; (2) when there is promise of increased trouble from compression of the ureters.

---

*On the Etiology of Pneumonia. Kohnhorn. Quarterly Reports of Hygiene. Centralblatt.*--Kohnhorn's conviction that croupous pneumonitis is an infectious disease and not due to cold, was gained from the manner of the appearance of that disease among troops in barracks at Wesel. His tables show that the majority of these cases did not fall in the cold months; further, that sudden changes of the weather, direction of the wind, quantity of ozone, falling of underground currents of water, did not account for the frequency of pneumonitis during the various months. Certain companies that had remained separated, but near others, would show many cases—both in summer and winter—while others would remain entirely free of a single case. The appearance of the disease could not be brought in connection with over-exertion in service, but rather dependent on certain barracks, into which it had insinuated itself endemically, sometimes mounting up to a local epidemic. Interesting was the fact that was proven on examination of the barracks, as to the cases of intermittent fever falling in the same years as pneumonitis cases: in eight years there were 308 cases of pneumonitis, and 305 cases of intermittent fever in about the same per cent. in the different barracks.

---

*Protoxide of Nitrogen as an Anæsthetic in Labor. A Klikowitch. Arch. f. Gyn., XVIII., p.* 81.—The author communicates his experience with this gas employed as an anæsthetic in twenty cases of labor. (Twelve cases are reported at length.) The duration of each pain was determined by keeping the hand on the uterus. In a few cases Schatz' tokodynamometer was employed for this purpose. K. comes to the following results; (1) laughing gas is harmless to mother and child; (2) birth is not delayed; (3) no pain at any period; (4) consciousness even at the height of anæsthesia; (he used a mixture containing oxygen); (5) no nausea, no vomiting, no headache; (6) the gas can be used continuously, no cumulative action arising therefrom ; (7) the presence of a physician to direct its employment is not absolutely necessary. As our author himself emphasizes, the price and non-portability of this anæsthetic are great drawbacks to its introduction in this manner, and for this purpose.

---

*Gumma of the Iris. Thomas Treitel. Wochenschrift, Berlin,* 1881, *No.* 28.—In the case of a young man, 20 years old, who was infected with syphilis, a gumma developed in the iris, and continued to grow in spite of inunctions and mercurial baths, until it filled out the anterior chamber. A superficial spot began to undergo cheesy degeneration, out of which a yellowish fluid ran along the posterior surface of the cornea, and was resorbed. Gradually, from this on, the tumor disappeared. As a soft cataract had developed, the patient had to submit himself to an operation, and, later, the pupil being closed, iridectomy was performed, the patient receiving from the two operations tolerable sight.

---

*Resection of the Stomach. Rydygier. Arch. fur Klin. Chiurgie, XXVI., p.* 731.—Rydygier advocates an incision in the linea alba, and the use of catgut ligatures, in resections of the stomach. The strong ground for the first is complete fixation of the duodenum, which is held with great difficulty over the abdominal integuments; for the employment of catgut instead of carbolized silk, as recommended by Billroth, the following reasons are given: In the intestinal canal even, silk is not resorbed, and the best disinfected silk may produce infection.

